# Supplementary material for: MetaRibo-Seq measures translation in microbiomes
Source: Nat Commun. 2020 Jun 29;11:3268. doi: 10.1038/s41467-020-17081-z (PMC7324362; doi:10.1038/s41467-020-17081-z)
Supplement: Supplementary file 10 — Supplementary Data 7 [file 41467_2020_17081_MOESM10_ESM.zip › File2/Confidence_High_Taxonomy/111087_out.krona.html]

Javascript must be enabled to view this page.

members
magnitude
magnitudeUnassigned
count
unassigned
taxon
rank

111087\_out

3

superkingdom
3
2

1239
phylum
3

186801
3
class

186802
3
order

186803
3
family

33042
genus
3

species
3

SRS014855\_contig\_number\_3884SRS015854\_contig\_number\_502SRS144714\_contig\_number\_19495
33043
